# Construyo e validado de cartilha para pais/cuidadores de crianzas com déficit de atenáo e hiperatividade*

**DOI:** 10.15649/cuidarte.3037

**Published:** 2023-12-21

**Authors:** Kely Vanessa Leite Gomes da Silva, Cristiana Brasil de Almeida Rebougas, Joáo Cruz, Paulo César de Almeida

**Affiliations:** 1 . Universidade Regional do Cariri, Crato, Brasil. E-mail: kelyvanessa@hotmail.com Universidade Regional do Cariri Universidade Regional do Cariri Crato Brazil kelyvanessa@hotmail.com; 2 . Universidade Federal do Ceará, Fortaleza, Brasil. E-mail: cristianareboucas@yahoo.com.br Universidade Federal do Ceará Universidade Federal do Ceará Fortaleza Brazil cristianareboucas@yahoo.com.br; 3 . Universidade da Integragáo Internacional da Lusofonia Afro-Brasileira, Redengáo, Brasil. E-mail: enfjcncruz@gmail.com Universidade Federal da Integração Internacional da Lusofonia Afro-Brasileira Universidade da Integragáo Internacional da Lusofonia Afro-Brasileira Redengáo Brazil enfjcncruz@gmail.com; 4 . Universidade Estadual do Ceará, Fortaleza, Brasil. E-mail: pc2015almeida@gmail.com Universidade Estadual do Ceará Universidade Estadual do Ceará Fortaleza Brazil pc2015almeida@gmail.com

**Keywords:** Transtorno do deficit de atengáo com hiperatividade, Tecnologia educacional, Estudos de validagáo, Educagáo em saúde, Enfermagem, Attention Deficit Disorder with Hyperactivity, Educational Technology, Validation Study, Health Education, Nursing, Trastorno por Déficit de Atención con Hiperactividad, Tecnología Educacional, Estudio de validación, Educación en Salud, Enfermería

## Abstract

**Introdujo:**

Os materiais educativos, como as cartilhas, sáo instrumentos que ajudam no cuidar e elucidam intervengoes relevantes e sensíveis ao contexto de saúde, especialmente no cuidado domiciliar a criangas desordem no neurodesenvolvimento.

**Objetivo:**

construir e validar cartilha para orientar pais e cuidadores de criangas com Transtorno de Déficit de Atengáo e Hiperatividade.

**Materiais e Métodos:**

estudo metodológico para construgáo e validagáo de cartilha por juízes e pelo público-alvo. Desenvolveu-se redagáo, designer e layout do material com posterior validagáo por juízes, considerando-se o índice de validade de conteúdo para análise dos dados.

**Resultados:**

cartilha composta por 13 temas e 32 páginas. A validagáo contou com 21 juízes de conteúdo obtendo índice de 0,89. Sete juízes em aparencia avaliaram e pontuou- se o índice de 0,91. 17 participantes do público-alvo avaliaram conteúdo geral com índice de 0,99. Após ajustes, o índice de legibilidade obteve percentual satisfatório de 54%.

**Discussáo e Conclusáo:**

O material construído retrata o cotidiano das famílias, apresenta ilustragoes em servigos de saúde e de educagáo e é sensível ao público estudado. A cartilha foi considerada válida quanto ao conteúdo e aparencia e apta para ser utilizada no cuidado da enfermagem a crianga com o transtorno, contribuindo para orientagáo adequada de pais e cuidadores.

## Abstract (en)

**Introduction:**

Educational materials, such as booklets, are tools that help in caregiving and elucidate relevant and sensitive interventions to the health context, especially in home care for children with neurodevelopmental disorders.

**Objective:**

To build and validate a booklet to guide parents and caregivers of children with attention deficit hyperactivity disorder.

**Materials and Methods:**

Methodological study for the construction and validation of a booklet by judges and the target audience. The material was written, designed, and laid out with subsequent validation by judges, considering the content validity index for data analysis.

**Results:**

Booklet composed of 13 themes and 32 pages. The validation had 21 content judges obtaining an index of 0.89. Seven judges in appearance evaluated and scored the index of 0.91. 17 participants from the target audience evaluated general content with an index of 0.99. After adjustments, the readability index obtained a satisfactory percentage of 54%.

**Discussion and Conclusion:**

The constructed material portrays the daily life of families, presents illustrations in health and education services and is sensitive to the studied audience. The booklet was considered valid in terms of content and appearance and suitable for use in nursing care for children with the disorder, contributing to adequate guidance for parents and caregivers.

## Resumen

**Introducción:**

Los materiales educativos, como los cuadernillos, son herramientas que ayudan en el cuidado y dilucidan intervenciones pertinentes y sensibles al contexto sanitario, especialmente en la atención domiciliaria de niños con trastornos del neurodesarrollo.

**Objetivo:**

Construir y validar un folleto para orientar a los padres y cuidadores de niños con Trastorno por Déficit de Atención e Hiperactividad.

**Materiales y Métodos:**

Estudio metodológico para la construcción y validación de un cuadernillo por jueces y público objetivo. El material fue redactado, diseñado y diagramado, y posteriormente validado por jueces, considerando el índice de validez de contenido para el análisis de los datos.

**Resultados:**

Folleto compuesto por 13 temas y 32 páginas. La validación contó con 21 jueces de contenido que obtuvieron un índice de 0,89. Siete jueces de apariencia evaluaron y puntuaron el índice de 0,91. 17 participantes del público destinatario evaluaron el contenido general con un índice de 0,99. Tras los ajustes, el índice de legibilidad obtuvo un porcentaje satisfactorio del 54%.

**Discusión y Conclusión:**

El material construido retrata la vida cotidiana de las familias, presenta ilustraciones en servicios de salud y educación y es sensible al público estudiado. El cuadernillo fue considerado válido en términos de contenido y apariencia y adecuado para uso en los cuidados de enfermería de niños con el trastorno, contribuyendo para la orientación adecuada de padres y cuidadores.

## Introdujo

O Transtorno do Déficit de Atengáo e Hiperatividade (TDAH) configura-se como uma desordem do neurodesenvolvimento, manifestando desatento, hiperatividade e impulsividade. Tal condigáo apresenta-se em tres subtipos principais: predominantemente desatento, predominantemente hiperativo-impulsivo e o tipo combinado^1^.

O TDAH representa até 50% das consultas psiquiátricas infantis e sua prevalencia varia de 2 a 12% da populagáo pediátrica mundial. Dentre as principais características ligadas ao transtorno observam- se: vigilancia auditiva, velocidade auditiva e visual, prudencia auditiva e distúrbios comportamentais oposicionistas^1^^-^^2^. Transtorno presente mais na infancia torna-se perceptível, principalmente, aos nove anos quando se inicia as dificuldades nos campos neurológico, psicopedagógico e fonoaudiológico, resultando na necessidade de cuidados específicos^3^. Manifestares clínicas relacionam-se as dificuldades na compreensáo de si, das pessoas e relacionamentos; ausencia de mentalizagáo do corpo; incapacidade de desenvolver e integrar experiencias ou atividades humanas; dificuldade na percepgáo objetividade/realidade e o comprometimento geral da personalidade^4^.

A partir da vivencia em Centro de Atengáo Psicossocial infantil (CAPSi) identificou-se despreparo da equipe de enfermagem em executar intervengóes adequadas e eficazes para a família de crianzas com este transtorno. Na vida de pais e cuidadores de criangas com TDAH a necessidade de orientagáo sobre o diagnóstico gera dificuldade na aceitagáo e no enfrentamento do transtorno^5^. Todavia, o envolvimento no tratamento facilita a compreensáo dos sinais e sintomas, edificam relacionamentos sólidos, desenvolve habilidades sociais e de autocontrole^3^^-^^6^.

A abordagem junto a família perpassa por atividades de educagáo em saúde promovidas pelos profissionais de equipe multidisciplinar e, dentre eles, o profissional de enfermagem^7^. O enfermeiro no cuidado a crianga com TDAH direciona-se ao acompanhamento do seu crescimento e desenvolvimento, promogáo e educagáo em saúde e o cuidado estendido a cuidadores como forma de promover a saúde da crianga^8^.

Por meio da sistematizagáo da assistencia de enfermagem, é possível, ainda, utilizar tecnologias durante as consultas de enfermagem. Isso favorece o processo criativo, auxilia o desenvolvimento de competencias, estimula o conhecimento e desenvolvimento de habilidades com autonomia, além de ser uma intervengáo terapeutica^9^. Para tal, torna-se necessária a capacitagáo com o uso de instrumentos/cartilhas que favoregam a promogáo da saúde^10^. As cartilhas se destacam pela construgáo lúdica, sucinta e de fácil entendimento, com parcela significativa de imagens^11^.

Construgáo e validagáo de materiais educativos embaseados no conhecimento científico por meio de interagáo dialógica entre pesquisador, especialistas e público alvo produz conteúdo próximo da realidade na qual se faz a intervengáo^12^. Para isso, faz-se necessário a apreciagáo dos atores imprescindíveis ao processo validativo^13^.

No caso de materiais impressos destinados a pais e cuidadores de criangas com TDAH, a cartilha educativa pressupóe o suporte nas demandas diárias com itens relevantes e sensíveis ao contexto em que será aplicado. Apesar da relevancia e aplicabilidade deste tipo de tecnologia para as equipes multiprofissionais, a literatura náo reporta nenhuma tecnologia voltada a este público revelando a necessidade de explorar a temática em questáo. Desse modo, objetivou-se construir e validar uma cartilha para orientar pais e cuidadores de criangas com Transtorno de Déficit de Atengáo e Hiperatividade.

## Materiais e Métodos

Estudo metodológico desenvolvido de fevereiro/2017 a maio/2018 nas etapas^14^: 1. Construyo da cartilha a partir da revisáo de literatura especializada, diagnóstico situacional e da produgáo das ilustragóes, layout, design e textos; 2. Validagáo da cartilha por juízes e público-alvo.

A etapa 1, de construgáo do conteúdo, iniciou-se por revisáo integrativa da literatura a partir da questáo norteadora: Quais as orientagóes de enfermagem devem ser repassadas para pais e cuidadores no cuidado a criangas com TDAH? As bases de dados selecionadas foram o Medical Literature Analysis and Retrieval System Online (MEDLINE), Literatura Latino-Americana e do Caribe em Ciencias da Saúde (LILACS) e Índice Bibliográfico Español en Ciencias de la Salud. Selecionou-se descritores do Medical Subject Headings (MsSH) e a estratégia de busca: caragivers AND Attention Deficit Disorder with Hyperactivity OR TDAH. A busca foi realizada de forma pareada por dois pesquisadores distintos. Estudos que contemplassem as necessidades quanto ao cuidado da família as criangas com TDAH e originais em portugues, ingles ou espanhol foram elegíveis. Após leitura de títulos e resumos, foram selecionados 23 artigos e extraídos os dados que corroborassem com a temática investigada.

A fim de subsidiar o diagnóstico situacional da etapa 1, foram entrevistados 25 pais/cuidadores de criangas com TDAH selecionados por conveniencia e que frequentavam os Centros de Atengáo Psicossocial infanto-juvenil (CAPSi) de dois municípios do interior cearense. Para se ter acesso aos cuidadores obteve-se autorizagáo dos CAPSi dos municípios para realizagáo das entrevistas, bem como solicitou-se informagóes das criangas usuárias dos servigos com diagnóstico ou hipótese diagnóstica de TDAH e de seus familiares-cuidadores. Os critérios de inclusáo foram: ser pai, máe ou cuidador de crianga com diagnóstico ou hipótese diagnóstica de TDAH, sendo a crianga usuária do servigo há pelo menos tres meses; participar das últimas consultas com as criangas usuárias do CAPSi e declarar ser cuidador no ambiente domiciliar dessa crianga. Nenhum cuidador foi excluído do estudo.

Após apresentagáo da pesquisa e aceite para participar da entrevista, foi solicitado a assinatura do Termo de Consentimento Livre e Esclarecido. Esta ocorreu em sala reservada nas dependencias dos referidos servigos, em data e horários reservados. Para condugáo da entrevista e auxílio na coleta de dados, a pesquisadora utilizou roteiro de sua autoria com itens sobre: O que é TDAH; quais os sinais e sintomas evidenciados; qual o tratamento e onde obte-lo; quais as atividades de cuidado sáo direcionadas as criangas. As entrevistas foram gravadas em áudio e transcritas na íntegra pela pesquisadora responsável. Após a gravagáo, os pais validaram o conteúdo. Para o encerramento desta etapa adotou-se o critério de saturagáo de dados, após a discussáo entre os pesquisadores. Utilizou-se a questáo: Quais as dificuldades e dúvidas no cuidado para com a crianga com TDAH? Os dados qualitativos foram analisados conforme a análise temática do conteúdo das falas em tres fases: pré-análise, exploragáo do material e interpretagáo^15^.

Em seguida, a análise da literatura e as respostas das entrevistas foram organizadas em temas como forma de subsidiar o conteúdo da cartilha: O que é TDAH; Sintomas de TDAH; como obter o tratamento; como é o tratamento; Orientagóes de cuidados a crianga com TDAH. Os textos e ilustragóes foram editados e diagramados por designer gráfico com auxílio dos programas Adobe Ilustrador CS3 para os desenhos e Adobe Indesign CS3 para o design; observando-se critérios de conteúdo, estrutura, layout, design, linguagem, contexto cultural/social adequado para os pais/cuidadores. Vale ressaltar que todas as imagens foram desenhadas para essa cartilha específica.

Finalizada a etapa de construyo, seguiu-se para a etapa 2, validagáo da cartilha. Destaca-se que foram selecionados e convidados uma média de 60 especialistas. Participaram 21 juízes de conteúdo, e sete de aparéncia, cujos critérios de participagáo evidenciassem experiencia profissional com assisténcia em saúde mental infanto-juvenil de no mínimo dois anos e publicado na temática; titulagáo de doutorado, mestrado ou residencia além de especializado na área de saúde mental, psiquiatria, neuropediatria, neurociéncias, Psicopedagogia; orientado em trabalho de graduado ou pós-graduado na área Saúde Mental ou TDAH; e docencia em disciplinas de saúde mental ou afins.

Para juízes de aparéncia, priorizaram-se aqueles com desenvolvimento ou publicado de trabalhos sobre construgáo e validado de tecnologias educacionais em saúde; com titulagóes de doutorado, mestrado ou especializado na área de Educado ou Educado em Saúde; e orientado de trabalhos de graduagáo ou pós-graduagáo ou, ainda, atuassem no ensino de Tecnologias educativas em saúde.

Os 21 juízes de conteúdo e os sete de aparéncia foram selecionados por conveniéncia e, de forma complementar, pela estratégia bola de neve e que concordassem em participar da pesquisa. Os juízes foram identificados a partir da plataforma Lattes e utilizou-se a busca simples com as palavras-chave: Saúde Mental, Psiquiatria, TDAH, infancia e tecnologia educacional de forma combinada, a fim de refinar os critérios de selegáo.

Os juízes receberam o TCLE, o formulário para caracterizagáo dos dados de identificagáo como também avaliaram a versáo digital da cartilha. Juízes de conteúdo avaliaram os critérios de objetivos, conteúdo, relevancia, no total de 20 itens. Já os juízes de aparéncia avaliaram os critérios de linguagem, ilustragóes, layout, motivagáo e cultura totalizando 18 itens e estes consideraram os pressupostos para construgáo e eficácia de materiais educativos^16^. Posteriormente, calculou-se o índice de legibilidade de Flesh que considera satisfatório o valor igual ou maior de 40%. Após as avaliagóes destes, procedeu- se a revisáo ortográfica e gramatical e, em seguida, calculou-se o índice de legibilidade Flesch por meio do programa ReGra®.

Para validagáo com o público-alvo e finalizagáo da etapa 2, foi entregue a cartilha impressa e um questionário com dados socioeconómicos e avaliagáo de objetivos, organizado, linguagem, aparéncia, motivagáo e adequagáo cultural, conforme recomenda a literatura^16^. Para a aparéncia, utilizou-se a Suitability Assessment of Materials (SAM)^17^. Após contato com os servigos dos CAPSi, a pesquisadora obteve acesso a 17 cuidadores de crianga com TDAH, usuários do CAPSi há pelo menos trés meses, que aceitaram participar do estudo.

Utilizou-se o índice de validade de conteúdo (IVC), obtendo-se o escore pela soma das opgóes “concordo” ou “concordo totalmente”, dividido pelo número total de respostas dos juízes. Considerou- se aceitável o índice maior ou igual a 78%^18^. Tanto no grupo de juízes como no dos pais e cuidadores, a validagáo ocorreu em etapa única, sendo julgadas as sugestóes pertinentes dos avaliadores e realizadas as modificagóes solicitadas. Desse modo, esta pesquisa considerou IVC acima de 0,78 como adequado. Os dados foram compilados e analisados utilizando o programa Statistical Package for the Social Sciences, versáo 20.0, licenga n° 10101131007. O conjunto de dados foi armazenado no DataSet Mendley Data^19^.

Pesquisa aprovada pelo comité de ética da Universidade Regional do Cariri sob parecer número 2.112.622/2017, atendendo a resolugáo 466/2012 do Conselho Nacional de Saúde.

## Resultados

A cartilha contemplou o conteúdo da síntese da revisáo bem como o resultado da consulta aos pais/ cuidadores de crianzas com TDAH durante o diagnóstico situacional. A construyo foi finalizada por designer gráfico que desenvolveu editoragáo, layout e diagramagáo. As evidencias científicas sintetizadas pela revisao versaram sobre conceito; sintomas; comorbidades; impacto nas esferas pessoal, familiar e social; tratamento; e abordagem familiar para um cuidado efetivo.

Nas entrevistas, 25 pais/cuidadores deram sugestóes enfatizando as lacunas de conhecimentos bem como o anseio em saber lidar com a crianga no ambiente familiar, social e escolar. Dentre os assuntos elencados, destacaram-se conhecimento da patologia, disciplina da crianga, suporte da escola no aprendizado e como obter ajuda social no que diz respeito as necessidades financeiras pelas quais a família vivencia nesse contexto.

Para iniciar a etapa 2, de validagáo da cartilha, foram apresentados os seguintes tópicos: Objetivos; Aplicagáo; Apresentagáo; O que é TDAH; Sintomas de TDAH; como obter o tratamento; Como é o tratamento; Orientagóes de cuidados a crianga com TDAH; Sua ajuda no tratamento é importante; Protegáo e apoio a crianga com TDAH; Dicas de sites e leituras sobre TDAH e Mensagem final.

Os 21 juízes de conteúdo residiam em Sáo Paulo, Bahia, Pernambuco e Ceará. Predominou o sexo feminino com 76,2%; 38,1% eram especialistas e mestres; 57,2% enfermeiros; 47,6% tinham entre tres a nove anos de formagáo e experiencia na educagáo; todos com publicagáo na temática e 47,6% com experiencia em estudos de validagáo. Nesse grupo haviam também médicos (9,5%), psicólogos (23,9%), terapeuta ocupacional e psicopedagogo (4,7%), respectivamente.

Na validade de conteúdo, obteve-se o IVC por critério que variou entre 0,72 a 0,90 conforme destacado na Tabela 1. Para fins de avaliagáo, os objetivos referem-se aos itens O1 a O5; quanto a estrutura e a apresentagáo tem-se os itens C1 a C9, e os itens de R1 a R3 sobre a relevancia.

**Tabela 1:** Valor do índice de validade de conteúdo por itens individuais do conteúdo por juízes em Saúde Mental. Fortaleza, CE, Brasil, 2018

Itens	I- IVCt
O1-Os objetivos sao coerentes com as necessidades do público-alvo	0,90
O2- A cartilha auxilia no cuidado a crianza com TDAH	0,93
O3-A cartilha é capaz de promover reflexao	0,84
O4- A cartilha pode promover mudanza de comportamento e atitude	0,85
O5- A cartilha pode circular no meio científico da área de saúde mental	0,86
C1- A cartilha educativa é apropriada para orientado dos pais e cuidadores quanto aos cuidados a crianza com TDAH	0,89
C2- A cartilha esclarece dúvidas	0,84
C3- A cartilha ressalta a importancia do conteúdo	0,85
C4- As mensagens estao apresentadas de maneira clara e objetiva	0,87
C5- As informales apresentadas estao científicamente corretas	0,83
C6- Os conteúdos sao variados e suficientes para atingir os objetivos da cartilha	0,72
C7- Existe uma sequencia lógica do conteúdo proposto.	0,82
C8-A divisao dos títulos e subtítulos do material sao pertinentes	0,89
C9- As ideias chaves (trechos em destaques) sao pontos importantes e merecem destaque	0,90
R1- Os temas retratam aspectos-chave que devem ser reforjados durante as consultas	0,87
R2-O material permite a transferencia e generalizares do aprendizado a diferentes contextos (hospitalar e domiciliar)	0,80
R3- A cartilha propóe ao aprendiz adquirir conhecimentos para realizar o cuidado com crianza com TDAH.	0,84

*tIVC: índice de validade de conteúdo. IVCgeral: 0,89*

Em relagáo ao item C6 que avaliou se os conteúdos sao variados e suficientes para atingir os objetivos da cartilha, este obteve IVC de 0,72 e foi revisado para prosseguir com a etapa subsequente, validagáo de aparéncia.

Observou-se, neste estudo, uma avaliagáo significante para o IVC em todos os dominios da cartilha. Portanto, optou-se por considerar pertinente as sugestóes dos juízes no quesito do conteúdo ser suficiente e variado para atingir os objetivos da cartilha e, desse modo, náo houve necessidade de passar por nova avaliagáo de juízes.

Os sete juízes de aparéncia constituíram-se de mulheres (85%), com mais de 50 anos (42%), doutoras e enfermeiras com publicado e experiencia em estudos de validagáo, Tabela 2.

**Tabela 2:** Valor do índice de validade de conteúdo por itens individuais do conteúdo por juízes especialistas em aparéncia. Fortaleza, CE, Brasil, 2018

Itens	I- IVCt
L1-As informajóes apresentadas sao claras e compreensíveis ao se levar em considerado o nivel de experiencia do público-alvo (pais e cuidadores de crianjas com TDAH)	0,94
L2- O estilo da redajao corresponde ao nível de conhecimento do público-alvo (pais e cuidadores cuidadores de crianjas com TDAH)	0,94
L3- As informajóes estao bem estruturadas em concordancia a ortografia	0,83
L4- A escrita utilizada é atrativa	0,86
11- As ilustrajóes utilizadas sao pertinentes com o conteúdo do material	0,91
12- As ilustrajóes estao expressivas e de fácil entendimento.	0,89
13- O número de ilustrajóes está suficiente.	0,89
14. As legendas das ilustrajóes estao adequadas e auxiliam o leitor a compreender a imagem	0,83
LY1- A apresentajao da cartilha está atrativa e bem organizada.	0,94
LY2- O conteúdo está apresentado com letra em tamanho e fonte adequados.	0,86
LY3- O tipo de letra utilizado facilita a leitura do material.	0,83
LY4- As cores dos textos sao adequadas e facilitam a leitura	0,94
LY5- A disposijao do texto está adequada.	0,86
LY6- O papel da impressao do material está apropriado	0,63
LY7- O número de páginas está adequado.	0,89
M1- O conteúdo desperta interesse para a leitura.	0,94
M2- O conteúdo está motivador e incentiva o leitor a prosseguir a leitura	0,94
C1- O material está apropriado ao nivel sociocultural do público-alvo proposto.	0,94

O item LY6 o qual avaliava a qualidade da impressáo do material obteve IVC de 0,63 com a menor avaliagáo. Essa avaliagáo baixa está relacionada ao envio da cartilha para avaliagáo apenas no formato digital, sem que os avaliadores tivessem analisado o material impresso. Os outros itens obtiveram um IVC que oscilou entre 83% a 94%. Desse modo, a cartilha é clara, adequada e relevante para o cuidado de criangas com TDAH.

Após validagáo de aparéncia, aplicou-se o índice de legibilidade de Flesh, na cartilha completa, obtendo-se fácil compreensáo (54%). A etapa de validagáo com o público-alvo constituiu-se de 17 pais/cuidadores provenientes de cidades do interior do Ceará com a prevaléncia de máes 16 (94,2%), entre 20 e 29 anos (35,3%), uniáo estável (88%), ensino fundamental I completo (35,3%) e católicas (88,3%). Quanto ao IVC geral dos critérios pelos pais/cuidadores obteve-se o valor de 0,99 confirmando a aprovagáo em aparéncia e conteúdo pelo público-alvo.

**Figura 1:**
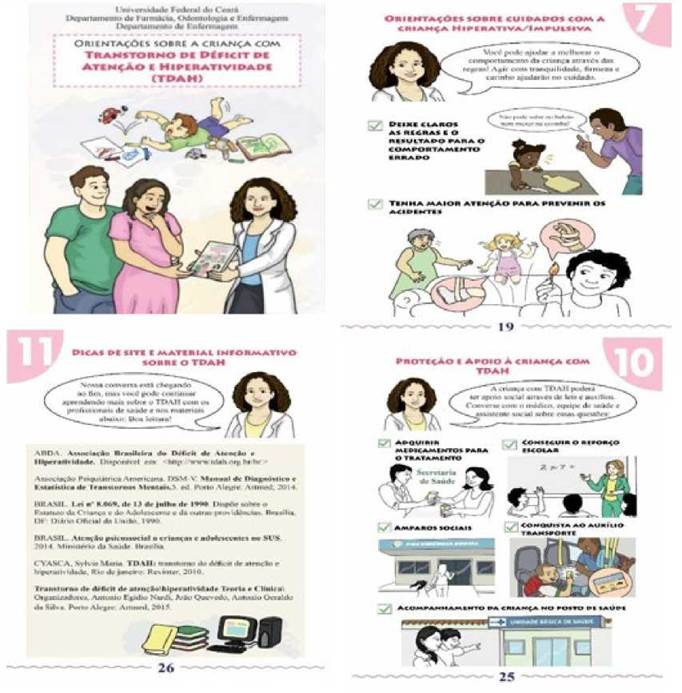
Capa e páginas da cartilha "Orientagóes sobre a crianza com transtorno de déficit de atengáo e hiperatividade (TDAH) ", Fortaleza, CE, Brasil, 2018

O público proferiu opinióes satisfatórias quanto a cartilha. Assim, a versáo final foi composta por 32 páginas, 13 critérios e intitulada: Orientagóes sobre a crianga com transtorno de déficit de atengáo e hiperatividade (TDAH), conforme a Figura 1.

## Discussao

No processo de construgáo dos materiais educativos foi importante reconhecer o contexto do público ao qual o material se destina. Nesse sentido, outra pesquisa de validagáo mostrou que ao inserir a abordagem participativa de forma coletiva, a validagáo permite produzir um instrumento claro e compreensível^12^. Assim, esta cartilha incluiu a participado efetiva dos cuidadores tanto para o diagnóstico situacional como para validado. O uso de tecnologias educativas validadas atribui maior qualidade ao cuidado no processo de ensino-aprendizagem além de melhorar a comunicado na assisténcia em saúde. Reforja, ainda, a confiabilidade das orientagóes apresentadas pelo profissional educador, contribuindo para a promogáo da saúde do público-alvo^20^.

A cartilha obteve IVC geral entre os juízes de 0,89, apresentando validade em aparéncia e conteúdo. Outros estudos IVC geral maior que 80% também empregaram validado com conteúdo e aparéncia em que os juízes eram profissionais de saúde e aplicaram as mesmas categorias avaliativas (objetivo e relevancia)^12^ e (conteúdo, linguagem, layout, ilustragóes, motivado e cultura)^21^ o que corrobora com a forma de validagáo do estudo em tela. Ressalta-se que a construgáo e validagáo de tecnologias educacionais permite a aproximagáo entre teoria, prática e público-alvo com a temática investigada, conferindo-lhe rigor metodológico^22^.

Quanto a validagáo de conteúdo pesquisas na enfermagem apontaram IVC de 0,93^23^^-^^24^. No caso deste estudo, obteve-se escore de 0,83. Neste quesito é importante refletir sobre a clareza e sequéncia lógica das informagóes da cartilha que receberam menores IVCs, respectivamente. Contudo, o domínio ainda foi significante e representativo para o estudo o que pode apontar, para novas pesquisas, se há aprendizagem dos cuidados com uso da cartilha.

A relevancia obteve IVC de 0,83 no estudo em tela. Em outros estudos obteve-se IVC maior de 0,95^13^^,^^21^^,^^24^, todavia outra pesquisa obteve IVC de 0,68^21^. Esta categoria é responsável pela generalizagáo da cartilha aos diferentes contextos e também sua importancia social^13^^,^^21^. O resultado de 0,83 deve-se a área de avaliagáo a aplicabilidade do estudo no contexto hospitalar visto que seu desenvolvimento se dá em um servigo de atengáo secundária e sáo necessários ajustes para aplicabilidade em outro nível de atengáo.

Destaca-se que neste estudo o item avaliado pelos juízes de conteúdo como objetivos obteve IVC de 0,85. Em outros estudos, houve aproveitamento de IVC maior que 0,95^13^^,^^21^^,^^24^ Este quesito é responsável pela adequabilidade da proposta do estudo e engloba fatores referentes a usabilidade, disseminagáo e promogáo da saúde^9^. No caso do estudo em tela atribui-se menor IVC (0,84) apenas quanto a reflexáo da cartilha o que pode ser justificável se relacionado a escolaridade do público alvo.

A linguagem e o layout aproximam a cartilha do público estudado, desta forma torna-se uma área difícil para atingir bons escores devido a necessidade de aproximagáo, sensibilidade cultural e adequabilidade^21^. Por isso, ressalta-se que no estudo em tela, faz-se necessário aprimorar a linguagem a medida que o público tenha contato com a cartilha e aprimore seus conhecimentos sobre TDAH a partir dela.

A validagáo dos critérios objetivos, ilustragóes, layout, motivagáo e cultura sáo essenciais para instrumentos em saúde pois denotam a aplicabilidade na prática com textos em linguagem compreensível, claro, atrativo e em sequéncia lógica, especialmente quanto aos objetivos, conteúdo, linguagem e relevancia^21^^-^^22^.

Em relagáo as ilustrares o IVC deste estudo foi de 0,85. Quanto a este critério, os estudos ressaltam que sáo elementos fundamentais na organizado e divulgado do material ao passo que aproximam da realidade. O layout também é um elemento essencial no processo de validado e neste estudo obteve IVC de 0,87. Estudos ressaltam que este critério é responsável pelo campo visual, suas fontes, cores e formas e aumenta a atratividade do instrumento^21^^,^^23^.

No que concerne a motivado e cultura, entende-se que os materiais devem ser interessantes e sensíveis a realidade do público alvo, contudo além de preservar a cientificidade necessária para tal instrumento deve estar alinhado a cultura do grupo pesquisado reconhecendo suas preferencias^22^^-^^23^. Neste estudo a avaliagáo foi satisfatória obtendo-se valor de IVC 0,94.

Materiais educativos em que o público-alvo participa e avalia quanto ao conteúdo, linguagem e aparencia torna-se estratégia interessante para que o material se torne adequado para a populado a qual se destina. Consequentemente, o material alcanzará o seu objetivo quando for aplicado durante a prática profissional. Portanto, recomenda-se que em todo estudo metodológico seja valorizado a etapa de avaliagáo pelo público-alvo^25^. Observa-se uma avaliagáo relevante para o IVC em todos os critérios da cartilha. O grau de legibilidade da cartilha aqui validada foi considerado fácil, o que corrobora com outros estudos de validado^12^^-^^13^^,^^22^^,^^26^^-^^27^. Demostra-se, portanto, clareza no texto, ilustrares adequadas e alta credibilidade cientifica.

A cartilha torna-se uma alternativa capaz de promover educado em saúde, facilitando a autonomia e encorajando as capacidades^21^. Estudo revelou que estratégias educativas sáo mais efetivas do que atividades tradicionais, aumentando a adesáo e promovendo autocuidado^26^.

Cartilhas devem ser de acordo como contexto estudado, próxima ao público e com conteúdo redigido na realidade de vida^18^. Para tal, esta cartilha obteve a imersáo no contexto de vida, possibilitando a identificado dos pais e fomentando o conhecimento das demandas por eles abordadas em resposta as suas necessidades.

O material construido retrata o cotidiano das familias, apresenta ilustragóes em servidos de saúde e de educado. Os individuos que participam de abordagem educativa tornam-se mais propensos a adotar novos comportamentos pela relagáo de confianga e proximidade^11^.Além disso, há imagens próximas dos textos facilitando a compreensáo dos pais/cuidadores.

As informagóes elucidam dúvidas inerentes ao cuidado e as relagóes social, familiar e educacional da crianga com TDAH por meio da educagáo em saúde. O texto e as ilustragóes confirmam o conteúdo teórico e as orientagóes, o que facilita a comunicagáo visual, aproxima os leitores e favorece o entendimento de pessoas em diferentes niveis de escolaridade^13^^,^^27^^-^^28^.

Consequentemente, materiais educativos auxiliam no processo de sensibilizagáo para o cuidado de si e do outro, pois as populagóes necessitam de materiais úteis ao cotidiano de criangas, pais e cuidadores que convivem com o TDAH^29^. Há escassez de pesquisas sobre esse tema, especialmente na educagáo em saúde^12^. Assim, alerta-se para a necessidade de investimentos na produgáo dos materiais educativos para ampla divulgagáo fisica e digital^13^.

Ressalta-se, ainda, que para escolha dos juizes em conteúdo optou-se por selecionar diferentes profissionais quanto a sua formagáo (médicos, enfermeiros, psicólogos, terapeuta ocupacional, psicopedagogo) com o intuito de buscar a contribuigáo significativa de cada área de atuagáo, tendo em vista que o tratamento e cuidado de criangas com TDAH requer a colaboragáo de uma equipe multiprofissional. Dessa forma, um olhar multidisciplinar torna-se essencial no processo avaliativo^23^. Quanto a contribuido significativa do profissional de enfermagem no processo de validado, estudo ressalta que este permite que o produto seja ancorado nas características da formado e atuagáo profissional^13^.

Reflete-se a atuagáo do enfermeiro ao contemplar o direcionamento de cuidados para com a crianza e o esclarecimento de dúvidas e suporte no acompanhamento para os pais. O uso de tecnologias educativas contribui efetivamente para educado em saúde e empoderamento da familia no ámbito do cuidado da crianza^8^.

Com a cartilha, o enfermeiro pode direcionar os cuidados para a crianza e esclarecer dúvidas dos pais. O uso de tecnologias contribui efetivamente para educado em saúde e empoderamento da familia no ámbito do cuidado a crianza^10^. Por isso, a apresentado de materiais por profissionais capacitados favorece o desenvolvimento cientifico da profissáo, impacta nas estratégias de promodo e prevendo de agravos em doengas, facilita o autocuidado e eleva a qualidade de vida^24^^,^^26^.

Disponibilizar materiais educativos já validados sáo úteis para padronizar as informagóes quanto ao cuidado realizado além de possibilitar a consulta tanto para os profissionais quanto para os pais/ cuidadores. Além disso, quando utilizadas no contexto familiar, reduzem as dificuldades no cuidado e na vida diária visto que o TDAH dificulta o acompanhamento do crescimento e desenvolvimento da crianga.

### Limitagdes do estudo

Como limitagóes do estudo, identificou-se a amostragem por conveniencia; número de máes e cuidadores de criangas com TDAH para validagáo de aparencia; greve dos servigos de saúde onde ocorreu a coleta de dados; escassas pesquisas direcionadas a temática proposta; número de pesquisadores para coleta de dados havendo a necessidade de estender o período do estudo e a impossibilidade de comparagáo intergrupo da cartilha com outro método de ensino.

### Contributes para a prática

Equipe de Enfermagem e multiprofissional do CAPSi como da Atengáo Primária em Saúde pode incluir esta cartilha no cuidado a crianga e sua família para melhor entendimento e compreensáo do TDAH. Almeja-se que, uma vez implementada nos servigos, especialmente no CAPSi, a cartilha possa subsidiar a promogáo da saúde por meio da prática do enfermeiro.

## Conclusao

A cartilha foi considerada válida quanto ao conteúdo e aparencia e apta para ser utilizada no cuidado da enfermagem a crianga com o transtorno, contribuindo para orientagáo adequada de pais e cuidadores. A tecnologia educativa construída e validada neste estudo, por meio da avaliagáo dos juízes especialistas em conteúdo de saúde mental, material educativo impresso e populagáo alvo, objetiva estimular o aprendizado sobre o TDAH e o cuidado a crianga pelos pais e cuidadores por meio da educagáo em saúde dos atores envolvidos.

Acredita-se que esta tecnologia educacional contribui para o cuidado do enfermeiro no campo da saúde mental e de outros profissionais da saúde, visto que as inovagóes tecnológicas vem transformando as práticas de ensino, possibilitando avangos científicos frente a problemática abordada.

Espera-se que a utilizagao desta tecnología possibilite melhoria da aprendizagem acerca dos cuidados com a crianga com TDAH. Além disso, espera-se maior articulado entre os profissionais da saúde e da educagao, especialmente na área da saúde mental infanto-juvenil, despertando mudangas de paradigmas quanto as práticas profissionais vigentes. Utilizar diferentes estratégias de cuidado em saúde torna-se imprescindível para que os pais e cuidadores de criangas com este déficit saibam identificar as necessidades das criangas e sejam empoderados para elevar a qualidade de vida de toda a família.
